# Lamotrigine-Induced Acute Pancreatitis

**DOI:** 10.7759/cureus.33135

**Published:** 2022-12-30

**Authors:** Emad Elmusa, Muhammad Waleed Raza, Ahmad Muneeb, Ameer Hamza, Mujtaba Butt

**Affiliations:** 1 Internal Medicine, Hospital Corporation of America (HCA) Florida Orange Park Hospital, Orange Park, USA; 2 Gastroenterology, Borland Groover Clinic, Jacksonville, USA

**Keywords:** drug-induced pancreatitis, mri, ct, ultrasound, valproic acid, pancreatitis, lamotrigine

## Abstract

Drug-induced pancreatitis is a rare phenomenon. Therefore, diagnosis requires ruling out more common etiologies of acute pancreatitis. The majority of research on drug-induced pancreatitis is from case reports. Only a limited number of drugs have been definitively established to induce pancreatitis. Lamotrigine is used in both bipolar and epilepsy. Lamotrigine is currently weakly identified to induce pancreatitis. We present a case of lamotrigine-induced pancreatitis. Extensive workup ruled out other major causes of pancreatitis-including alcohol. We aimed to show lamotrigine can be a causative drug of acute pancreatitis.

## Introduction

There are approximately 2,30,000 cases of pancreatitis every year requiring hospitalization [1]. There are multiple etiologies of pancreatitis, and drugs are a rare cause. The majority of research regarding drug-induced pancreatitis relies heavily on case reports [2]. In 2020, 213 unique drugs were described as causes of acute pancreatitis [1]. Importantly, all drugs implicated in inducing pancreatitis are categorized based on the strength of evidence. Karch and Lasagna categorize drugs as definite, probable, and possible [2]. Wolfe et al. categorize drugs into classes Ia-IV [1]. Lamotrigine has been described only once to be a causative agent of pancreatitis and is referenced as class IV [1]. We present a case of lamotrigine-induced pancreatitis. Other causes of pancreatitis were ruled out. The patient was followed for approximately one year after discharge and reported no episodes of pancreatitis after cessation of lamotrigine.

## Case presentation

**Figure 1:**
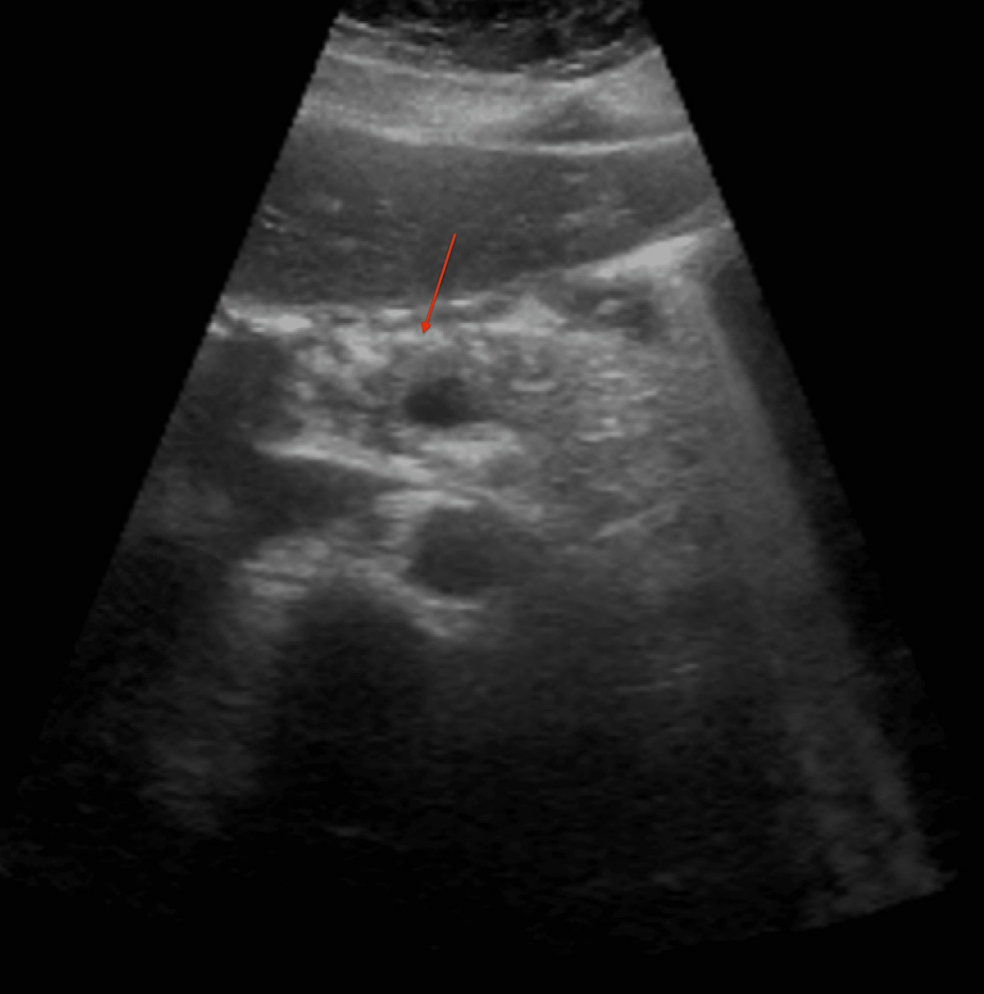
Ultrasound with hypoechoic pancreas consistent with acute pancreatitis.

A 35-year-old female with a past medical history significant for bipolar type II presented with a chief complaint of epigastric abdominal pain for four days. The patient denied alcohol use or history of gallstones. The patient reports compliance with home medications amphetamine/dextroamphetamine 30 mg twice daily, sertraline 50 mg daily, and lamotrigine extended-release 25 mg daily. The patient states that she was started on lamotrigine two weeks ago. The patient was previously on sodium valproate for mood stabilization. However, this medication was discontinued because the patient was diagnosed with valproate-induced pancreatitis twice, with the last time being six months ago. The patient denied recent trauma, family history of pancreatitis, or recent fevers. On presentation in the emergency department, vitals revealed a temperature of 36.8°C, heart rate of 72 beats per minute, respiratory rate of 18 breaths per minute, blood pressure of 135/92 mmHg, and pulse oxygen saturation of 98%. On physical examination, the patient’s abdomen was tender to palpation in the epigastric area but soft and non-distended. Labs were significant for a lipase of 931 units/L (reference: 73-393 units/L). Aspartate transaminase, alanine transaminase, alkaline phosphatase, total bilirubin, and calcium were within normal range. Serum quantitative alcohol was negative. Ultrasound revealed no gallstones but did show hypoechogenicity of the pancreas consistent with acute pancreatitis (Figure 1).

**Figure 2:**
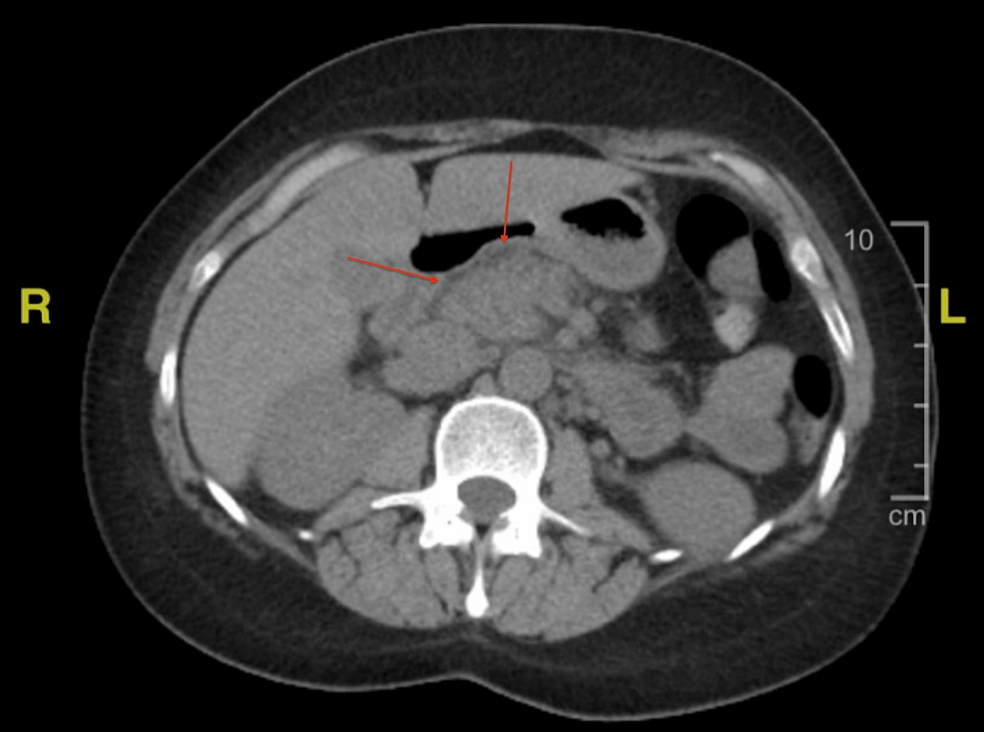
Upper abdominal CT scan without contrast shows peripancreatic edema consistent with acute pancreatitis.

Computed tomography (CT) of the abdomen and pelvis without contrast was also performed, which showed peripancreatic edema along the head and body consistent with acute pancreatitis (Figure 2). The patient was given intravenous isotonic fluids, and analgesics were given as needed. The patient’s home lamotrigine was discontinued.

**Figure 3:**
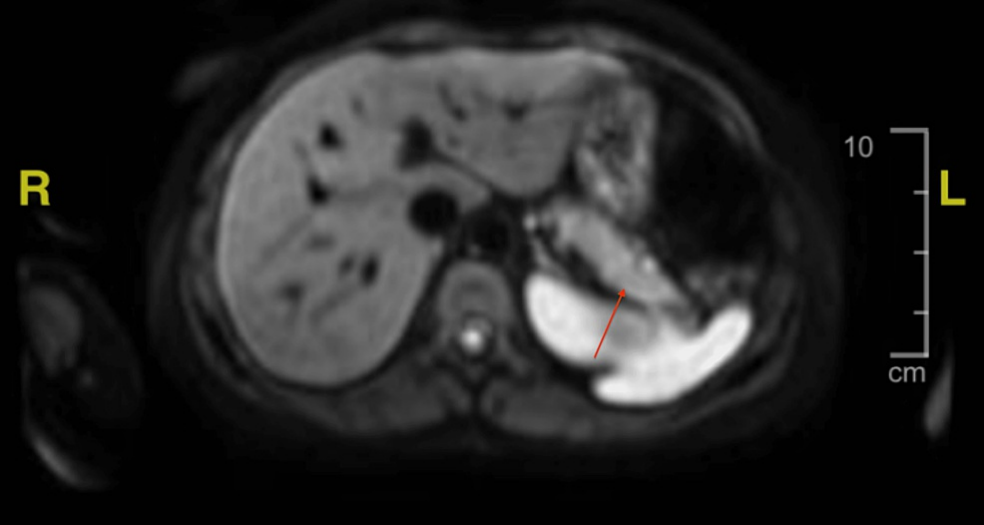
Diffusion restriction within the distal body and pancreatic tail of the pancreas is noted on upper abdominal MRI.

Gastroenterology evaluated the patient and recommended magnetic resonance imaging (MRI) of the pancreas to evaluate for other etiologies of the patient’s pancreatitis. MRI demonstrated diffusion restriction within the distal body and tail of the pancreas consistent with acute pancreatitis (Figure 3). Further workup revealed a triglyceride level of 120 mg/dL (reference: <150 mg/dL), carbohydrate antigen 19-9 of 22.6 units/mL (reference: 0.0-30.9 units/mL), IgG of 707 mg/dL (reference: 586-1602 mg/dL), IgG1 of 388 mg/dL (reference: 248-810 mg/dL), IgG2 of 163 mg/dL (reference: 130-555 mg/dL), IgG3 of 55 mg/dL (reference: 15-102 mg/dL), IgG4 of 36 mg/dL (reference: 2-96 mg/dL), and anti-nuclear antigen was negative.

The patient did well and was discharged on hospital day 3. The patient was instructed to stop taking lamotrigine and to follow up outpatient with psychiatry and gastroenterology. The patient was contacted 11 months after presenting to our institution, and she reported no other episodes of pancreatitis after stopping lamotrigine.

## Discussion

Drug-induced pancreatitis is rare compared to other causes of pancreatitis-accounting for only 0.1-2% of acute pancreatitis cases [2]. Drug-induced pancreatitis is typically mild and self-limiting [1]. Drugs are only considered when other etiologies are ruled out [2]. Therefore, gallstones, alcohol, hypercalcemia, hypertriglyceridemia, pancreatic cancer, pancreatic anatomical variants, biliary causes, viral causes, trauma, genetic causes, autoimmunity, and other drugs must be ruled out [1,2]. Interestingly, the majority of research on drug-induced pancreatitis is based on isolated case reports [2]. Karch and Lasagna categorized drugs inducing pancreatitis as definite, probable, and possible [2]. Only 31 drugs were designated as definite causes of acute pancreatitis [2]. Wolfe et al. categorized drugs inducing pancreatitis into classes Ia to IV, and only 45 drugs were designated as class 1a [1]. Of note, valproic acid is identified as a class 1a drug [1].

The pathophysiology of drug-induced pancreatitis is not well understood. There are theories about certain drugs. For example, metronidazole is thought to exert a direct toxic effect on the pancreas, while angiotensin-converting enzyme inhibitors are thought to cause angioedema of pancreatic ducts [2]. Additionally, because drug-induced pancreatitis is rare, it is important to also identify at-risk individuals who may be more prone to developing pancreatitis secondary to drug exposure. This includes females, pediatrics, and the elderly [2]. Additionally, underlying diseases can also be risk factors for developing drug-induced acute pancreatitis. This includes inflammatory bowel disease, diabetes mellitus, liver disease, immunologic disorders, and renal dysfunction [1].

Lamotrigine is used as a mood stabilizer in patients with bipolar disorder [3]. Lamotrigine side effects include nausea, vomiting, diarrhea, irritability, headache, insomnia, dizziness, ataxia, tremor, drug reaction with eosinophilia and systemic symptoms (DRESS), and Stevens-Johnson syndrome [3,4]. Lamotrigine overdose is associated with convulsions, hypokalemia, encephalopathy, and QRS widening [5]. Lamotrigine is described once as a possible cause of pancreatitis, but it is regarded as class IV evidence [1]. The case describes a patient who overdosed on lamotrigine, but the patient also used alcohol [4]. 

We presented a 35-year-old female patient in whom pancreatitis was diagnosed based on clinical symptoms, lipase elevation greater than three times the normal, and image findings consistent with pancreatitis on ultrasound, CT, and MRI. All other causes of pancreatitis were ruled out with exception of viral and genetic testing. Given the temporal relationship of lamotrigine use and acute pancreatitis, as well as no further episodes of pancreatitis following cessation of lamotrigine, lamotrigine is the most probable cause of the patient’s pancreatitis. Re-exposure was deemed inappropriate for this patient as other drugs were available for mood stabilization.

## Conclusions

Drug-induced pancreatitis is rare and evidence is primarily supported by case reports. We present a case report of a patient with lamotrigine-induced acute pancreatitis in which the majority of other causes are ruled out. As discussed, there are individuals who are more at-risk to develop drug-induced pancreatitis. Our patient likely fits into this category based on gender, unknown individual genetics, and prior episodes of valproate-induced pancreatitis. Our goal is to present this case in order to offer evidence that lamotrigine is a probable cause of pancreatitis.
